# The structure–function relationships and physiological roles of MnSOD mutants

**DOI:** 10.1042/BSR20220202

**Published:** 2022-06-17

**Authors:** Rosalin Bonetta Valentino

**Affiliations:** Barts and the London, School of Medicine and Dentistry, Queen Mary University of London, Victoria, Malta

**Keywords:** mutation, structural characterization, superoxide dismutases

## Abstract

In this review, we focus on understanding the structure–function relationships of numerous manganese superoxide dismutase (MnSOD) mutants to investigate the role that various amino acids play to maintain enzyme quaternary structure or the active site structure, catalytic potential and metal homeostasis in MnSOD, which is essential to maintain enzyme activity. We also observe how polymorphisms of MnSOD are linked to pathologies and how post-translational modifications affect the antioxidant properties of MnSOD. Understanding how modified forms of MnSOD may act as tumor promoters or suppressors by altering the redox status in the body, ultimately aid in generating novel therapies that exploit the therapeutic potential of mutant MnSODs or pave the way for the development of synthetic SOD mimics.

## Introduction

**Scheme 1:**
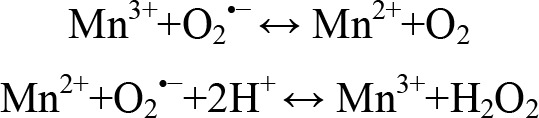
MnSOD dismutation

MnSODs act as the first line of defence against reactive oxygen species (ROS) by catalyzing the dismutation of two molecules of superoxide to oxygen and hydrogen peroxide (H_2_O_2_), using a cyclic reduction and oxidation reaction of the active site metal (Scheme 1).

**Figure 1:**
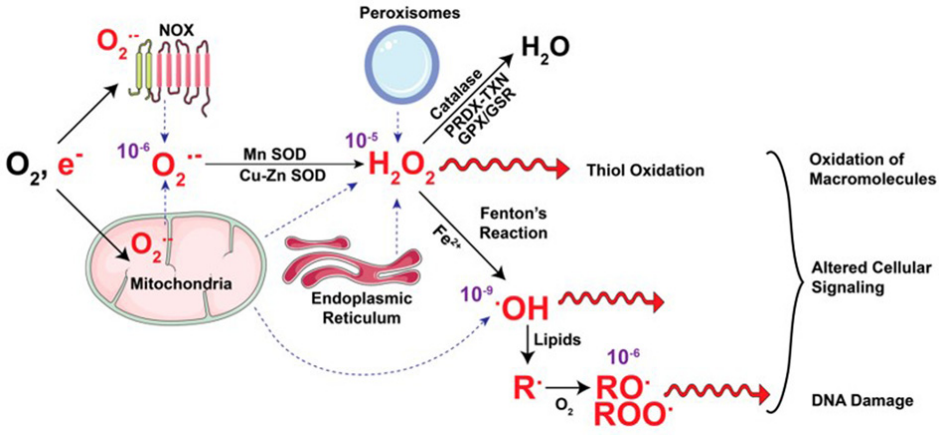
The fate of ROS The electron transport chain (ETC) and NADPH oxidases (NOX) take up oxygen to generate superoxide. Superoxide is dismutated by MnSOD and Cu-ZnSOD to generate H_2_O_2_. Oxidation of cysteine residues of peroxiredoxin (PRDX) and thioredoxin (TXN) proteins converts H_2_O_2_ to water. H_2_O_2_ is also converted to water by the glutathione peroxidase-glutathione reductase (GPX-GSR). In the presence of Fe^2+^ ions, H_2_O_2_ is converted to hydroxyl radicals (**^•^**OH) via the Fenton reaction. H_2_O_2_ and **^•^**OH radicals oxidize macromolecules, altering cellular signaling and oxidatively damaging DNA. This leads to gene mutations. The **^•^**OH radicals start lipid oxidation of alkyl (R**^•^**) groups, which are oxidized to alkoxyl (RO**^•^**) and peroxyl (ROO**^•^**) radicals. The dotted blue arrows show the source of ROS while purple numbers show the half-life of these radicals (in seconds). Adapted from Purohit et al., 2019 [1].

Superoxide is a strong oxidizing agent whereby high levels lead to a cascade of reactions (Figure 1) that cause damage to biological molecules including nucleic acids, proteins and other biomolecules including lipids.

**Figure 2:**
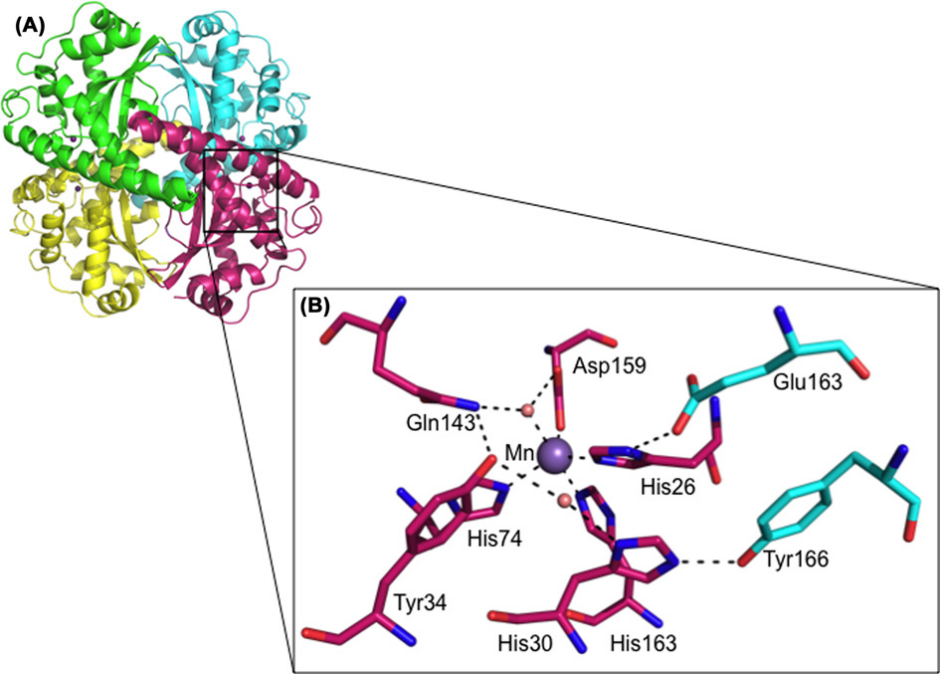
The quaternary and active site structure of human MnSOD (**A**) Human MnSOD (PDB code: 1LUV). Each monomer is illustrated by a different color and together the four subunits of MnSOD form the tetramer. (**B**) The active site structure of human MnSOD depicts the hydrogen-bonding network from the direction of access to the substrate. The manganese cofactor and the water molecules are depicted as purple and red spheres, respectively. Residues from different subunits, are colored pink and cyan, respectively. Adapted from Bonetta (2018) [7].

MnSOD in humans is constituted by a homotetramer, with each of the four subunits containing an active site with a manganese as a cofactor (Figure 2). The metal is coordinated by the His26, His74, His163, Asp159 residues and an oxygen-containing molecule (depicted by a red sphere in Figure 2B), which could be either a water or a hydroxide [2]. The amino acids that coordinate directly with manganese are referred to as ‘inner sphere’ residues [3]. The next layer of amino acids in the active site of the enzyme are referred as ‘outer sphere’ residues and are essential to perform the dismutation reaction. The amino acids which are crucial in the enzyme catalysis mechanism and make up the outer sphere include His30, Tyr34, Phe77, Trp78, Trp123, Gln143, Trp161 and Glu162 from the adjacent subunit.

The substrate diffuses and enters the active site via His30 and Tyr34, where it coordinates with the manganese cofactor [4]. The amino acids Tyr34, His30 and Gln143 form a hydrogen-bonded network to relay protons to the manganese cofactor in order to carry out successful electron transfer (dashed lines, Figure 2B) [5,6].

Excessive amounts of superoxide are involved in the pathogenesis of many diseases that include cancers, neurodegenerative diseases and cardiovascular disorders [8,9]. Therefore, understanding structural and functional polymorphisms of the MnSOD gene is important to determine what constitutes an adequate level of ROS within the cell for minimal biomolecular and nucleic acid damage and effective signaling. This review also describes how a recombinant form of MnSOD that is characterized by the presence of a leader peptide allows MnSOD to enter cells selectively. The latter enables this recombinant form of MnSOD to be used therapeutically in chemotherapy or radiodiagnosis of cancer by coupling radioactive substances or chemotherapic drugs to the MnSOD leader peptide [10]. Low MnSOD expression has been associated with different types of cancer. Conversely, overexpression of MnSOD has been linked with preventing cancers in humans, suggesting MnSOD as a tumor suppressor [11]. The activity of MnSOD also appears to affect the tumor-promoter or tumor-suppressor effects of the protein [12].

The mutagenesis work done on MnSODs in prokaryotes and eukaryotes in the past two decades was carried out to understand the elements in the protein which determine its quaternary structure as a dimer or tetramer, to recognize the importance of the generally conserved trigonal bipyramidal geometry of the active site, as well as the features of catalysis affecting the MnSOD mechanism (Table 1). Mutations of specific residues in MnSOD served to shed light on how certain amino acids are essential for hydrogen bonding to maintain the dimeric structure and hence, enzyme activity and thermal stability. Different physicochemical properties of the twenty amino acids, which include features such as hydrophobicity, polarizability and the average mass of the amino acids have been included in Table 2 to better appreciate how altering amino acids in MnSODs, may lead to different structure–function relationships in MnSOD mutants. Other mutations found closer to the manganese cofactor were found to either alter the trigonal bipyramidal active site structure or disrupt the hydrogen bonding network required for electron transport to successfully eliminate superoxide radicals. Particular amino acid substitutions were also found to impact the metal selectivity and thermostability of these enzymes.

**Table 1:** Kinetic data and PDB codes related to MnSODs and the respective mutants

MnSOD	*k*_1_ (nM^−1^ s^−1^)	*k*_2_ (nM^−1^ s^−1^)	*k*_3_ (nM^−1^ s^−1^)	*k*_4_ (nM^−1^ s^−1^)	PDB code
Human^a^	1.5	1.1	1.1	120	1LUV
Y34A^a^	0.25	<0.02	0.38	330	1ZSP
Y34N^a^	0.14	<0.02	0.15	200	2P4K
Y34H^a^	0.07	<0.02	0.04	61	1ZTE
Y34V^a^	0.15	<0.02	0.15	1000	1ZUQ
Y34F^b^	0.55	<0.02	0.46	52	1XIL
H30N^c^	0.21	0.40	0.68	480	2GDS
H30Q^c^	0.57	0.79	0.79	200	1LUW
H30V^c^	∼0.005	∼0.03	0.16	0.7	1NON
E162D^d^	0.36	0.13	0.21	40	3C3T
E162A^d^	0.06	0.05	0.09	30	3C3S
F66A^e^	0.6	0.5	0.7	82	2QKA
F66L^e^	0.7	0.8	0.2	40	2QKC
W123F^f^	0.76	<0.02	0.64	79	–
Y166F^d^	0.2	0.2	0.2	270	1PL4
W161A^g^	0.08	<0.01	0.37	180	1JA8
W161F^g^	0.3	<0.01	0.46	33	–
W161V^g^	–	–	0.27	265	–
W161Y^g^	–	–	0.20	130	–
W161H^g^	–	–	0.29	136	–
Q143A^h^	–	–	–	–	1EM1
H30N/Y166F^b^	0.1	0.1	0.1	440	1PM9
Y34F/W123F^f^	0.55	<0.22	0.46	52	1SZX
*S. cerevisiae^i^*	1.1–1.5	0.8	0.04–0.05	90–140	3LSU
*S. cerevisiae* Y34F^j^	–	<0.01	0.70	∼40	4E4E
*E. coli* ^e^	1.1	0.9	0.2	60	1D5N
*Deinococcus radiodurans* ^i^	1.2	1.1	0.07	30	2AW9
Y34F^k^	0.9	0.9	0.5	30	–
*Caenorhabditis elegans^l^*	1.2	0.5	0.5	300	3DC5
Q142H^m^	–	–	–	–	6ELK
H30N^n^	–	–	–	–	6S0D
*Staphylococcus equorum* S126C^o^					7DDW
*Staphylococcus aureus* G159L/L160F^p^					6QV8
F19I/G159L/L160F^p^					6EX5

The rate constants in the kinetic mechanism of numerous MnSODs and their mutants were adapted from Abreu and Cabelli [13]. No structures and hence PDB codes are available for the following MnSOD mutants: *Escherichia coli* E170A, G77Q/Q146A, K182R, A183P, K184R, L185P in *Saccaromyces cerevisiae* and *Candida albicans*, human Q143N and *Porphyromonas gingivalis* Q70G/A142Q. Data reproduced from ^a^Perry et al. [5], ^b^Hearn et al. [14], ^c^Hearn et al. [15], ^d^Quint et al. [16], ^e^Zheng et al. [17], ^f^Greenleaf et al. [18], ^g^Hearn et al. [3], ^h^Whittaker and Whittaker [19], ^i^Sheng et al. [20], ^j^Sheng et al. [21], ^k^Abreu et al. [22], ^l^Hunter et al. [23], ^m^Hunter et al. [24], ^n^Bonetta et al. [25], ^o^Retnoningrum et al. [26], ^p^Barwinska-Sendra et al. [27].

**Table 2:** Physicochemical properties of 20 amino acids

	*H*_1_	*H*_2_	*V*	*P*_1_	*P*_2_	SASA	NCI	MASS
**A**	0.62	−0.5	27.5	8.1	0.046	1.181	0.007187	71.0788
**C**	0.29	-1	44.6	5.5	0.128	1.461	−0.03661	103.1388
**D**	−0.9	3	40	13	0.105	1.587	-0.02382	115.0886
**E**	-0.74	3	62	12.3	0.151	1.862	0.006802	129.1155
**F**	1.19	-2.5	115.5	5.2	0.29	2.228	0.037552	147.1766
**G**	0.48	0	0	9	0	0.881	0.179052	57.0519
**H**	−0.4	-0.5	79	10.4	0.23	2.025	−0.01069	137.1411
**I**	1.38	-1.8	93.5	5.2	0.186	1.81	0.021631	113.1594
**K**	−1.5	3	100	11.3	0.219	2.258	0.017708	128.1741
**L**	1.06	-1.8	93.5	4.9	0.186	1.931	0.051672	113.1594
**M**	0.64	−1.3	94.1	5.7	0.221	2.034	0.002683	131.1986
**N**	-0.78	2	58.7	11.6	0.134	1.655	0.005392	114.1039
**P**	0.12	0	41.9	8	0.131	1.468	0.239531	97.1167
**Q**	−0.85	0.2	80.7	10.5	0.18	1.932	0.049211	128.1307
**R**	-2.53	3	105	10.5	0.18	1.932	0.049211	156.1875
**S**	−0.18	0.3	29.3	9.2	0.062	1.298	0.004627	87.0782
**T**	-0.05	−0.4	51.3	8.6	0.108	1.525	0.003352	101.1051
**V**	1.08	-1.5	71.5	5.9	0.14	1.645	0.057004	99.1326
**W**	0.81	−3.4	145.5	5.4	0.409	2.663	0.037977	186.2132
**Y**	0.26	-2.3	117.3	6.2	0.298	2.368	0.023599	163.1760

Properties include *H*_1_, hydrophobicity; *H*_2_, hydrophilicity; *V*, volumes of side chains; *P*_1_, polarity; *P*_2_, polarizability; SASA, solvent-accessible surface area; NCI, net charge of side chains; MASS, average mass of amino acid. Reproduced from Li et al. [28].

## Significance of the MnSOD quaternary structure

Wild-type and mutant forms of MnSODs in various organisms including *Escherichia coli* [19], *Saccaromyces cerevisiae*, *Candida albicans* [29] and more recently *Staphylococcus equorum* [30] were studied to understand the functional importance of the quaternary structure of MnSOD. Numerous studies highlight the role that the dimer interface plays in MnSOD stability and catalysis.

**Figure 3:**
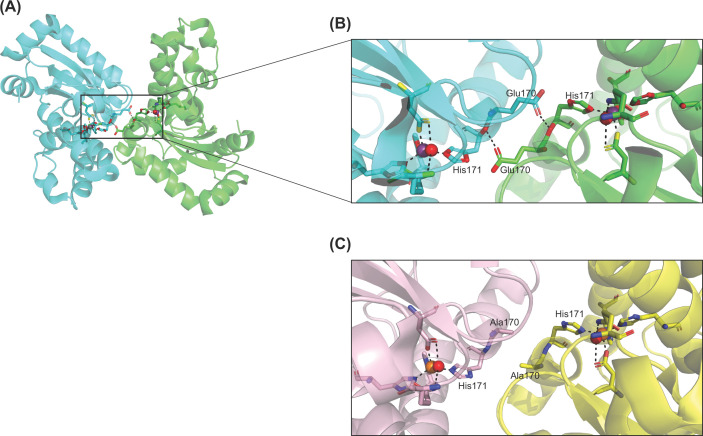
Quaternary structure of *E. coli* MnSOD (**A**) Dimeric structure of *E. coli* MnSOD (PDB code: 1D5N), with each subunit depicted by a different color (subunit A is colored cyan while subunit B is colored green). (**B**) Active sites of *E. coli* MnSOD depicting the inner sphere residues coordinating with the manganese cofactor and highlighting the hydrogen bonding formed between Glu170 and His171 between the two monomeric subunits. Manganese is depicted as a purple sphere. (**C**) The active sites of the E170A mutant using PyMol to reflect the disrupted hydrogen bonding and subsequent destabilization of the dimer structure. Metal specificity is altered in the E170A mutant; hence, iron is depicted as an orange sphere. The red spheres represent the water molecules coordinating with the metal cofactor. Hydrogen bonding is depicted by black dashes. All figures were generated using PyMol [31].

In *E. coli* MnSOD, Whittaker and Whittaker observed that the Glu170 of one monomer which forms a hydrogen bond with His171 in the other monomer, forms a double bridge at the dimer interface. The amino acid His171 is also one of the residues that coordinates directly with the manganese atom [30]. The Glu170A mutant protein resulted in a mixture of dimers and monomers, a change in metal specificity and the complete loss of catalytic activity [19] (Figure 3). Substitutions of Glu162 in human MnSOD, which is the counterpart of Glu170 in *E. coli* MnSOD, reduced the catalytic activity to 5–25% of that exhibited by the wild-type enzyme [18]. The human Y166F MnSOD mutant also demonstrated a great decrease in catalytic activity and a large unfolding transition at a lower *T*_m_ [14]. The substitution of Phe66 at the dimer interfaces of human MnSOD reduced product inhibition in the human enzyme, making it resemble *E. coli* MnSOD [13].

Further studies on point mutations at the dimer interface were performed on *S. equorum* MnSOD by Retnoningrum et al. In this case, substitutions were introduced to improve the dimer interface interaction. One substitution, L169W, was made at a region which was distant from both the active site as well as the dimer interface. Although the L169W mutant was dimeric like the wild-type, it had an altered monomeric form. However, enzyme activity was similar to that of the wild type [26]. Another mutant, N73F was generated, and the substitution was at the dimer interface. Conservation of the N73-F124 bonding in the structure of human MnSOD further supports the importance of these residues in maintaining the dimer structure. Residue S126 is also involved in interactions at the dimer interface [26]. Another substitution in *S. equorum* MnSOD, S126C (Table 1), was done by Retnoningrum et al., to reveal that 50–70% of monomers only actually formed the S-S bond. This flawed S-S bonding was probably caused by photolytic S-S bond breakage due to the neighboring tryptophan residue. The wild-type has S126 facing W163 and forming a hydrogen bond mediated by water with E164. Therefore, the presence of W163 and E164 are essential for enzyme activity. In fact, altering S126 to a cysteine lowered enzyme activity by ∼70%, highlighting the role of the S126 residue in the enzyme’s activity and stability. It is important to understand what contributes to dimer stabilization in MnSOD for potential therapeutic applications. Retnoningrum et al. mention that for example, having a MnSOD mutant with improved dimer stability in sunscreen would be useful, as the protein dimer would dissociate at higher temperatures.

Mutants in different yeast species were also generated and studied by Sheng et al. to understand the MnSOD quaternary structure better. Wild-type as well as mutant forms of *S. cerevisiae* MnSOD and *C. albicans* MnSOD with the substitutions at dimer interfaces were analyzed to understand the effect of these changes on their oligomeric states, resistance to pH, heat and denaturants. The mutations were K182R, A183P and K184R, L185P in *S. cerevisiae* and *C. albicans*, respectively.

Dimeric *C. albicans* MnSOD was observed to be much more susceptible to thermal or denaturant-induced unfolding than *S. cerevisiae* MnSOD, which is tetrameric. The residue substitutions at dimer interfaces triggered dimeric *C. albicans* MnSOD but not tetrameric *S. cerevisiae* MnSOD to dissociate into monomers. Sheng et al. therefore concluded that the tetrameric assembly strongly reinforces the dimer interface, which is crucial for MnSOD activity [21].

## Metal selectivity and enzyme activity in MnSOD mutants

Specific mutations in the MnSOD of different organisms have been observed to alter both metal specificity as well as enzyme activity. The *E. coli* MnSOD mutant E170A, was reported to contain only iron but did not demonstrate any form of superoxide dismutase activity (Figure 3). When observing spectroscopic properties, pH titration behavior and anion interactions, the E170A mutant strongly resembled the wild type FeSOD. However, reconstituting the E170A mutant with Mn(II) did not restore SOD activity even though Mn-reconstituted E170A MnSOD and wild type MnSOD were spectroscopically similar [19].

In human MnSOD, it was demonstrated that Q143 mutants alter metal specificity and active site redox potential. The Q143N mutant was also reported to have decreased catalysis [32,33] and a wider entry to its active site, allowing for the insertion of an additional water molecule [34]. Replacement of the glutamine by alanine (Table 1) kept the same enzyme fold, but it introduced two water molecules that took up the positions equivalent to the Oε1 and Nε2 of the missing glutamine. The substitution of glutamine by glutamic acid had a negligible effect, while the substitution of by lysine generated an enzyme that could not be purified [35].

In *E. coli* MnSOD the substitution of Q146 by glutamic acid (143 numbering in human MnSOD) produced an apoprotein, while the mutating Q146 to leucine or histidine reduced enzyme activity to less than 10%. The mutants had minimal structural changes when compared with the wild-type protein [36]. The Q146H mutant exhibited similar activities with either iron or manganese in the reconstituted enzyme, even though the levels of either iron or manganese were low [36].

**Figure 4:**
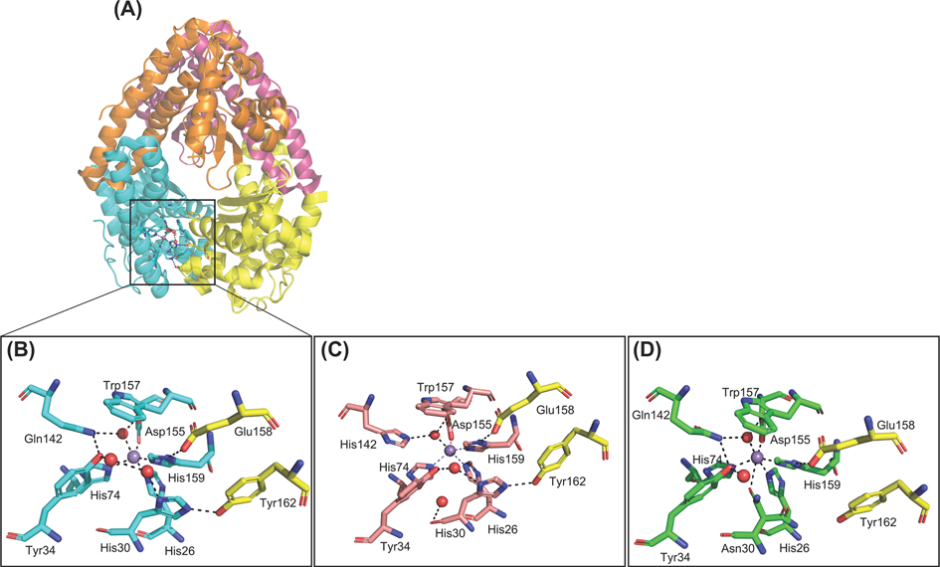
The quaternary and active site structure of wild-type and mutant *C. elegans* MnSODs (**A**) Tetrameric structure of wild-type *C. elegans* MnSOD (PDB: 3DC5). Each subunit is depicted by a different color. (**B**) The active site structure of wild-type MnSOD-3 depicting the hydrogen-bonding network from the direction of substrate access. (**C**) The active site of Q142H MnSOD-3 (PDB: 6ELK). D) The active site of H30N MnSOD-3 with N30 depicted as having two rotamers due to multiple conformations occupied by the amino acid (PDB: 6S0D) [25]. The manganese and the water molecules in B, C and D are shown as magenta and red spheres, respectively. All hydrogen bonding is depicted as black dashes. Residues from the adjacent subunits are colored yellow. Figure drawn using PyMol [31].

Changes in metal selectivity were also observed *Caenorhabditis elegans* MnSOD mutants, including the Q142H mutant (Figure 4). In this case, substituting glutamine 142 by histidine changed the metal selectivity from manganese to iron [24]. The latter substitution retained only 50% of the enzyme activity and became cambialistic as it exhibited enzyme activity with either iron or manganese. The substitution of histidine 30 by asparagine in *C. elegans* MnSOD also lead to a reduction in the incorporation of manganese and an increase in iron [25]. Although the H30N mutant took up iron spontaneously in addition to manganese, enzyme activity was reduced to 22% compared with the wild-type (Figure 4). Upon analyzing the H30N active site, Bonetta et al., reported no significant perturbations of the metal–ligand interactions, which include the interactions of His26, His74, His159 and Asp155 with the metal. However, further characterization of H30N by 285 GHz high-field electron paramagnetic resonance (HFEPR) confirmed the occurrence of a hexa-coordinated center and showed a change in the electronic structure of the enzyme.

Besides point mutations, double mutations were also introduced into the prokaryotes *Porphyromonas gingivalis* and *E. coli* SODs with the purpose of altering their metal specificities. In *P. gingivalis* SOD, the Q70G/A142Q mutations reduced the iron-supported enzyme activity and altered the ratio of Mn:Fe from 1.4 to 3.5 [37]. In *E. coli* MnSOD, the G77Q/Q146A mutations had manganese specific activity that was reduced to 71% and instead iron-supported SOD activity was introduced, which was not exhibited by the wild-type SOD [38].

In the *E. coli* Q142 mutants, although mutants that lacked a glutamine residue demonstrated the highest metal selectivity, they showed lower or negligible enzyme activity. However, double mutants G77Q/Q146A revealed increased enzymatic by 150% compared with wild-type MnSOD. However, in another study by Hunter et al., the same mutant was reported to have only 75% activity compared with the wild-type protein in a previous study [35]. This variability might be because of the different methodology employed in those two different studies. Hunter et al. reported some iron-supported activity (7%) in this mutant [35]. The most prominent change in the physical characteristics of the G77Q/Q146A double mutant was observed in temperature-sensitivity studies. In fact, a similar temperature-deactivation profile to that of the FeSOD wild-type enzyme was reported. This suggests that the position of the glutamine residue in the active site is responsible for this change, which is most likely due to its involvement in a hydrogen-bonding network that includes other residues and solvent molecules. An unexpectedly higher thermostability of the mutant G77Q MnSOD was also observed. Likewise the metal specificity of MnSOD isolated from *Staphylococcus aureus* was also studied. The author reported that when residue at position 159 was substituted metal specify was considerably affected.

The conversion of glycine 159 to leucine (G159L) showed a 10-fold increase its Fe-dependent activity compared with wild-type MnSOD, lowering its manganese-dependent activity by twice as much [27]. Although this mutant remained mainly manganese-specific, it exhibited increased cambialism. The reciprocal mutation generated in cambialistic SOD, whereby leucine was this time substituted by glycine at position 159 (L159G), lowered its iron-dependent activity >10-fold relative to wild type cambialistic SOD. On the other hand, it augmented manganese-dependent activity >3-fold. This resulted in a mutant with a MnSOD-like activity profile. Such results suggest that the amino acid at position 159 has a great effect on the ability of the *S. aureus* SODs to use iron and manganese. However, the role that this residue plays in affecting metal specificity needs to be further understood to explain the contrasting metal specificities of the two *S. aureus* SODs. Substituting the residue at position 160 also influenced metal specificity. The L160F mutant of MnSOD was similar to the G159L variant, resulting in a lower manganese-dependent activity and increased iron-dependent activity albeit to a lower extent than observed in the G59L mutant.

The G159L/L160F MnSOD double mutant showed a significant difference in metal utilization when compared with the wild-type or the single mutants (Table 1). The iron-dependent activity of the MnSOD double mutant was increased >20-fold relative to wild-type MnSOD, resulting in a highly cambialistic enzyme that displayed activity with manganese and with iron that differed only two-fold. The authors report that this highly cambialistic mutant enzyme demonstrated diminished activity with manganese, suggesting that the gain of cofactor flexibility compromises catalytic efficiency. Such results show how only two mutations in the secondary coordination sphere can switch MnSOD metal specificity [27].

The *S. aureus* MnSOD F19I/G159L/L160F triple mutant exhibited greater activity with manganese and less activity with iron than the G159L/L160F double mutant (Table 1). These studies demonstrate that positions 159 and 160 are important in regulating the reactivity of the metal cofactor. Mutations at these particular locations generated great effects on metal specificity. However, they did not lead to any changes to the protein backbone structure, as observed from their crystal structures. The structural arrangement of the metals’ primary ligand spheres as well as the substrate access channels and hydrogen-bonding networks that are essential for proton-coupled electron transfer remained the same [39]. Nonetheless, the mutations directly affected the electronic structure and electrochemical properties of manganese centers. In fact, HFEPR spectra of the wild-type *S. aureus* MnSOD and the mutants were different [40]. This is also in accordance with the findings of Bonetta et al. where both metal specificity as well as the electronic structure of the *C. elegans* H30N MnSOD mutant were altered, despite not undergoing any changes in the backbone structure [25]. Barwinska-Sendra et al. concluded that the altered metal specificity caused by the mutations is therefore, related to modified electronic structures and changes in the manganese reduction potential to become more catalytically active with iron [27].

## Kinetics of MnSOD mutants

**Scheme 2:**
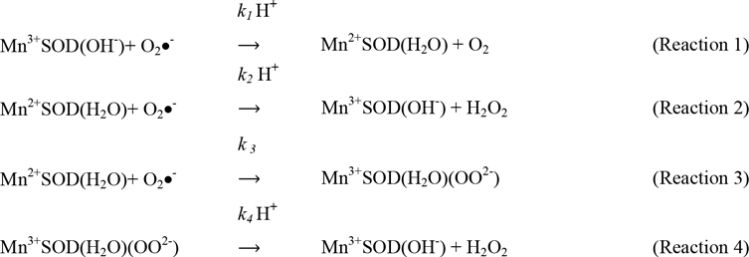
Mechanism of catalysis of MnSOD

The control of MnSOD catalytic features is achieved via the ability of the enzyme to eliminate superoxide by two different pathways, described by the McAdam scheme (Scheme 2) [41]. According to this scheme (Scheme 2), the catalytic cycle of MnSOD may be explained by four reactions that demonstrate that the dismutation of superoxide takes place via two pathways that are simultaneous. These two parallel pathways consist of the outer sphere (fast) pathway whereby the conversion of superoxide to H_2_O_2_ is immediate (Scheme 2, reactions 1 and 2), and the inner sphere (slow) pathway (Scheme 2, reactions 3 and 4) which involves the formation of the product–inhibited complex followed by the release of the H_2_O_2_.

In the first step of both the outer sphere and inner sphere pathways (Scheme 2, reaction 1), superoxide coordinates directly with the metal. Reduction of Mn^3+^ of the resting enzyme to Mn^2+^ by the superoxide is associated with the protonation of the hydroxide solvent ligand to H_2_O, with Gln143 being the proton source. During the fast outer sphere pathway, the second superoxide entering the active site bonds to the ligand water and to the outer sphere Tyr34 through a water molecule (reaction 2). This forms a proton relay that converts superoxide to H_2_O_2_ with the simultaneous oxidation of Mn^2+^ to Mn^3+^ and the generation of the resting form of the enzyme, Mn^3+^SOD(OH^−^) [42].

The slower inner sphere pathway leads when there are high superoxide concentrations. When the second superoxide interacts with the Mn^2+^ it forms a ‘dead-end’, reversible peroxy adduct, Mn^3+^SOD(H_2_O)(OO^2−^) (reaction 3) which inhibits the enzyme from finishing the reaction. H_2_O_2_ is formed and released via a mechanism that uses two protons, one that originates from the water ligand and another one that derives from the outer sphere hydrogen network and bulk water. During this process, the manganese ion is oxidized back to the resting Mn^3+^ state and is bound to the hydroxyl ligand.

The way this fine tuning is carried out has been the focus of numerous studies of MnSODs in various organisms; however, it is still not fully understood. The different crystal structures of MnSODs that were studied all show a great similarity around the active center. The vast mutagenesis work done on MnSODs to date, implies that the conditions leading to differences in the mechanisms exhibited by MnSOD proteins is not controlled by a single feature (Table 1). The *k*_2_/*k*_3_ gating ratio provides a value which indicates the preferred catalytic pathway used by the particular MnSOD under high superoxide concentrations and the tendancy for the formation of the peroxy adduct. One of the most interesting questions that arises from these kinetic studies is whether the difference in the *k*_2_/*k*_3_ gating ratio represents an evolutionary phenomenon without any functional significance or whether there is a purpose for the difference in the gating ratio between prokaryotes and eukaryotes. MnSODs with a *k*_2_/*k*_3_ ratio greater than 1, such as *E. coli* (4.5) and *Deinococcus radiodurans* [16] are less affected by product inhibition [17,22]. On the other hand, human and *C. elegans* MnSODs, have a gating ratio of 1 (Table 1) [5,23].

**Figure 5:**
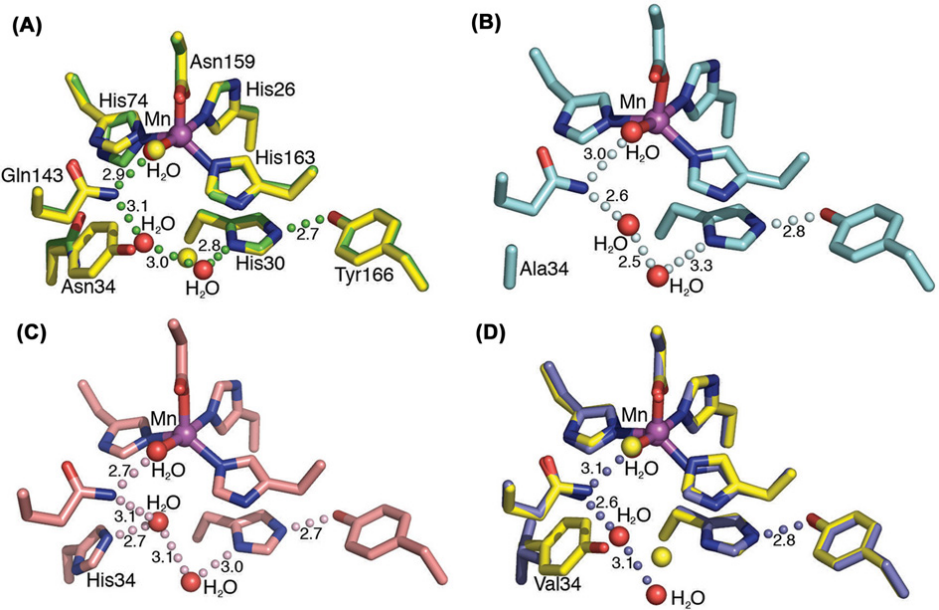
The human MnSOD active-site and mutations of Y34 (**A**) The superimposed active-site side chains of wild-type MnSOD and Y34N are colored yellow and green, respectively. Water molecules in wild-type MnSOD and Y34N are colored yellow and red, respectively. The Y34N hydrogen bond relay is illustrated by green spheres. Bond distances are annotated in angstroms. (**B**) The Y34A active site structure with the hydrogen bonding relay is colored pale cyan. (**C**) The Y34H active site structure, with the hydrogen bonding relay and distances is annotated in angstroms. (**D**) Superimposed active site structures of the human wild-type MnSOD and Y34V are colored yellow and blue respectively. The waters colored in red belong to Y34V while the hydrogen bond relay of Y34V MnSOD is illustrated by blue spheres. Bond distances are annotated in angstroms. Adapted with permission from Perry et al. (2009) [5]. Copyright 2009 American Chemical Society.

The kinetics of the second sphere residues Tyr34, His30, Gln 143, Tyr166 and Trp161 in human MnSOD mutants have been studied considerably to elucidate the catalysis mechanism and functional role of these residues with an interest in generating SOD with the fast catalysis demonstrated by the wild-type but with reduced product inhibition. The Tyr34 and His30 residues are highly conserved and are referred to as gateway residues as they are found to the entrance to the substrate funnel leading to the active site. The most studied Tyr34 mutant is Y34F [14,18,21,36,43–45]. At high levels of superoxide, human Y34F has a very low *k*cat and a negligible *k*_2_. In Y34F, catalysis occurred preferentially via the inner sphere pathway with the generation of the dead-end product. This is accompanied by a reduced rate of product dissociation (*k*_4_), suggesting that Tyr34OH in the hydrogen bonding network lowers the product inhibition and prefers the outer sphere pathway. Other Y34 mutants, namely, Y34A, Y34N, Y34H and Y34V (Figure 5) also show a decrease in *k*_1_ and *k*_2_ values, reducing catalysis in general, and increasing the level of the dead-end product. This supports the idea that *k*_2_ indicates the level of fast proton transfer to the hydrogen peroxide product.

In human MnSOD, the H30V mutation restricted access to the substrate funnel and interfered with superoxide binding, due to the steric impediment brought about by valine. Consequently, H30V was observed to have a reduced catalytic rate and higher product inhibition. The level of product inhibition varied among different His30 mutants. Whereas H30Q was observed to have a similar product inhibition to the wild-type, H30N was found to be the least product inhibition out of the His30 mutants [13].

The single mutations, H30N and Y166F, both resulted in a reduction of the *k*cat, *k*cat/*k*m and *k*1 values (Table 1). However, a greater reduction in catalysis was not observed for the double mutant H30N/Y166F, indicating cooperativity between the two residues [14]. The rate of product dissociation, *k*_4_, was noted to increase in the H30N and H30N/Y34F mutants but not in Y166F single mutation, suggesting the contribution of His30 or nearby water molecules in the rate of catalysis as well as the product inhibited complex stability.

Substitution of Q143 by alanine or asparagine introduced one or two water molecules in the active site cavity that complete the hydrogen-bonded network [33,34]. However, Q143A and Q143N mutants were still incapable of maintaining the catalytic activity and stability present in the wild-type human MnSOD.

Substitution of Trp161 by phenylalanine reduced catalysis to a third of the catalysis rate observed in the wild-type MnSOD. Product inhibition also increased substantially in Y166F. This suggests that although the conserved non-ligand residue contributes to the fastest level of catalysis, it is not fundamental. Nevertheless, it is involved in stabilizing the product-inhibited complex [46].

Studies were also performed on the catalysis of the Y34F mutant of *S. cerevisiae* by Sheng et al. Even though the catalytic function of the Y34F mutant of *Sc*MnSOD via the prompt protonation pathway is affected negatively, the rate of protonaton off is increased. This may be due to a water molecule responsible for the detected six coordinate intermediate product. Although the water molecule within bonding distance to the metal was not reported in the structure, a water molecule may move in to replace the hydroxyl group of the mutated tyrosine. A water molecule in an equivalent location was identified in both human MnSOD [5] and *E. coli* MnSOD [47] Y34 mutants which demonstrate a fast level of protonation.

## The role of H30N MnSOD mutants in human tumor cell lines

The effect of the human and *C. elegans* H30N mutant on human tumor cell lines was investigated *in vivo* and *in vitro*, respectively. Davis et al. report how the human H30N MnSOD mutant enzyme with a higher *k*_4_ and quicker hydrogen peroxide production than the wild-type MnSOD exhibited striking *in vivo* effects [48]. The transient overexpression of H30N in human cell lines increased cell viability in a TNF-mediated apoptosis model. On the other hand, stable overexpression of this mutated enzyme brought about a strong anti-proliferative effect in the transformed cells. Although human H30N MnSOD induced cytoprotection was not observed in the stable H30N transformed cell lines, which could mean that TNF cytotoxicity does not merely depend on the increase in superoxide, the authors demonstrate how overexpression of mitochondrial-targeted catalase can rescue the H30N MnSOD anti-proliferative phenotype. This established a critical regulatory effect for hydrogen peroxide produced during MnSOD catalysis. Additionally, *in vivo* overexpression of the human H30N mutant resulted in a striking inhibition of tumor growth in an animal model of tumorigenesis, further highlighting the significance of MnSOD and MnSOD-derived hydrogen peroxide production in the control of cellular growth [48].

On the other hand, the exogenous addition of the *C. elegans* H30N MnSOD mutant to K562 cells, resulted in increased cell viability of the K562 cells. One inference that may be derived from the modified activity of H30N as well as the signaling properties of hydrogen peroxide would be that the catalytic mechanism of the H30N mutant generates hydrogen peroxide product at a level which stimulates cell growth [25].

## MnSOD variants associated with disease

Since cells that lack mitochondrial MnSOD activity are incapable of surviving, [49], very few polymorphisms of MnSOD are linked to pathologies. The most extensively studied MnSOD polymorphism is the A16V single nucleotide polymorphism in codon 16 of the mitochondrial targeting leader sequence that substitutes the alanine residue (GCT) to valine (GTT) and is the ninth amino acid from the first amino acid of the mature protein [50–52].

The MnSOD variant with valine at position 16 results in a conformational change of the secondary structure of the mitochondrial target of the MnSOD protein from an α-helix to a β-sheet [53]. This prevents the successful import of MnSOD through the TIM23 mitochondrial import channel [54]. MnSOD is subsequently degraded by the proteosome and the MnSOD activity is reduced further via RNA degradation. Conversely, the alanine variant, has an α-helical secondary structure that enables mitochondrial import, allowing MnSOD to be imported efficiently within the mitochondrial matrix and therefore, leading to higher SOD activity [54].

The correlation of cancer with the A16V polymorphism is not a simple one as it seems to have contrasting effects in different cancer types [55–57]. Various studies have found links between the valine form of the MnSOD gene and increased cancer risk [58–60]. The most substantial correlation involves the greater risk in lung cancer associated with patients that are homozygous for Val/Val [61,62]. Bergman et al. report how the MnSOD valine allele may be used as an indicator of risk for early onset of breast cancer, since the onset of the disease was found to be higher in carriers that were homozygous for Val/Val [60]. On the other hand, many studies revealed the alanine form as being associated with a higher cancer risk, such as esophageal cancer [63], colorectal cancer [64] and cervical cancer [65]. Other studies did not find any significant association between this polymorphism and cancer risk [66–69].

Such studies show that the amount of cellular ROS determined by MnSOD activity levels may either contribute to or prevent tumorigenesis. The variant phenotype is affected also by the patient’s antioxidant status. In the case of prostate cancer, malignancy was worse in Val/Ala patients with low selenium levels and Ala/Ala patients with low intake of vitamin E [70,71]. Such observations were demonstrated in the meta-analysis by Wang et al. which revealed how the polymorphism phenotype was exacerbated by an unstable antioxidant status [71].

Valenti et al. found that the A16V polymorphism influences the risk of cardiomyopathy related to iron overload and may act as an iron toxicity modifier [72]. Gamarra et al. reported that the A16V MnSOD polymorphism is linked to the onset of Familial Alzheimer’s disease in carriers of the ApoE4 allele [73]. Pournourali et al. suggest that the MnSOD A16V polymorphism may be related to a risk of female infertility in northern Iran [74]. More recently, it was reported by Liu et al. that the A16V polymorphism may also be one of the genetic determinants for PCOS in Chinese women [75].

Other polymorphisms one that converts isoleucine 58 to threonine (I58T) [76], and a polymorphism whereby leucine is converted to phenylalanine (L60F) [77]. Upon transfection into the MCF-7 breast cancer cell line, the I58T variant demonstrated reduced tumor suppressor activity [78]. The L60F variant was identified in Jurket human T leukemia cells and contributes to their malignancy. Isoleucine 58 essentially positioned in the N-terminal hairpin loop that takes part in the interactions at the tetrameric interface of the quaternary structure. Therefore, replacing isoleucine by threonine was observed to destabilize the tetrameric interface [79] and reduce the protein's thermostability.

## Liposarcoma-derived recombinant (LSA) MnSOD

The LSA MnSOD isoform has been found in the mitochondria, the rough endoplasmic reticulum and secretion vesicles in liposarcoma cells [10,80]. Upon being secreted, it retains its leader peptide constituted by 24 amino acids and enters tumor cells in a selective manner. It is capable of exerting cytotoxic effects as long as cells possess an estrogen receptor and express low levels of catalase [81].

Mancini et al. observed that the recombinant form of LSA-type MnSOD (rMnSOD) exhibits cytotoxic properties, which occur due to induced Bax-dependent apoptosis [81]. This cytotoxic effect is reduced in cells having high catalase activity, suggesting that hydrogen peroxide is involved in the cell death mechanism. The leader peptide resembles that of the mitochondrial precursor MnSOD which is expressed in normal cells having the Ala16 polymorphism. However, this leader peptide has a glutamine replacing glycine at position 12 and a hydrophobic leucine replacing the polar serine at position 13. When the leader peptide, which acts as a molecular carrier was conjugated to cisplatin, it provided an efficient delivery into various types of cell but triggered fast apoptosis only in tumor cells [82]. Borrelli et al. distinguished a segment of six amino acids with the sequence AVCGTG that interacted with the estrogen receptor and suggest that this segment was responsible for entry of the leader peptide into the cell [83]. Therefore, Borrelli et al. found that by using the leader peptide as a molecular carrier, considerably lower amounts of antitumor agents could be administered to patients with cancer. This leads to increased antitumor action and this approach could transform generically molecules (such as cisplatin) into antitumor drugs that are specific [82,84]. For this reason, Borrelli et al. suggest that the recombinant MnSOD with the leader peptide conjugated to cisplatin should be considered as a novel antitumor agent.

Damiano et al. found that rMnSOD could prove to be useful when treating or preventing ischemic damages to the kidney brought about by lengthy immune suppressive therapies with antitumor drugs or Cyclosporin-A [85]. Pisani et al. also demonstrated that rMnSOD may protect the kidneys via X-ray contrast media [86]. More recently, Cataldi et al. demonstrated that rMnSOD also protects the midbrain cells considerably from radiation-induced damage, generating a drastic up-regulation of neutral sphingomyelinase gene as well as protein expression [87].

## Post-translational modifications of MnSODs

**Figure 6:**
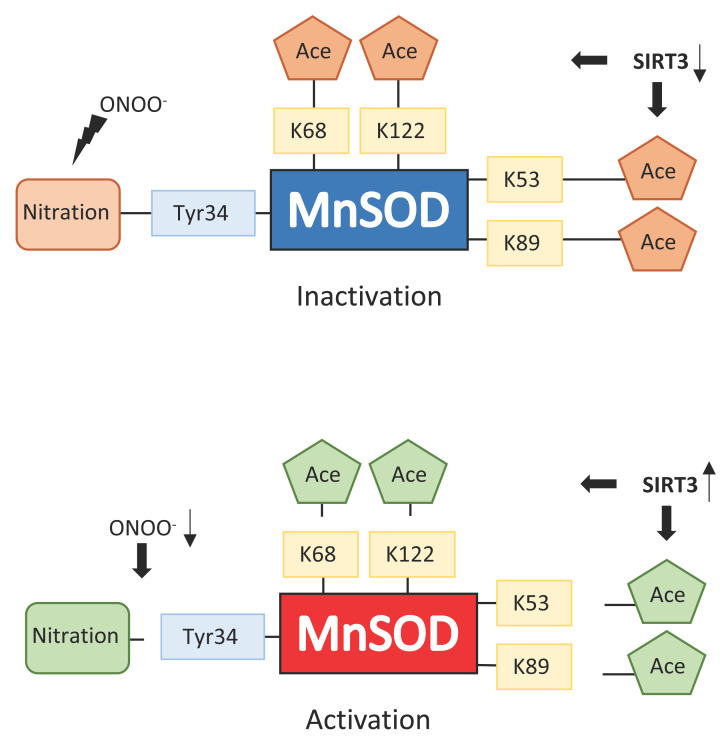
MnSOD activity regulation via translational modifications Peroxynitrite encourages the MnSOD inactivation by nitrating Y34 at the enzyme active site. MnSOD acetylation at amino acids K68, K122, K53 and K89 induces MnSOD inactivation as well. Reduced levels of peroxynitrite and activation of SIRT3 lead to the reactivation of MnSOD via deacetylation or denitrification of the targeted residues. Adapted from Kitada et al. using CC-BY license [110].

Acetylation regulation of human MnSOD at particular lysine residues, which includes K122 (precursor numbering) [88] and K68 (precursor numbering) is maintained by Sirt3 (NAD-dependent deacetylase sirtuin-3, mitochondrial) [89]. Acetylation status of MnSOD, particularly K68, controls the detoxification activity of ROS, besides providing a link between metabolic stress and mitochondrial pathways that provide a balance in metabolism (Figure 6). Reduced SOD activity due to acetylation leads to increased mitochondrial superoxide levels. Irradiation, nutrient depletion as well as oxidative stress bring about deacetylation of MnSOD by Sirt3. Therefore, ROS scavenging capacity increases within the mitochondria under adverse conditions. The positively charged lysine is suggested to attract superoxide anions into the electrostatic funnel, directing them to the active site via electrostatic facilitation as proposed by Irwin Fridovich [90,91].

Zhu et al. explain how their biochemical experiments that used the MnSOD-K68Q Ac-mimic or K68-Ac (MnSOD-K68-Ac), imply that these monomers function as peroxidases, which is different from the conventional MnSOD superoxide dismutase activity. Cells expressing MnSOD^K68Q^ exhibited resistance to tamoxifen, while cells that were chosen for tamoxifen resistance demonstrated higher levels of K68-Ac and monomeric MnSOD [92]. Such results implicate the presence of a MnSOD-K68-Ac metabolic pathway for tamoxifen resistance, tumor progression and carcinogenesis. Hence, Zhu et al. show MnSOD in the homotetrameric and monomeric forms functions as a superoxide dismutase and a peroxidase, respectively, whereby the homotetramer acts as a tumor suppressor, while the monomer resulting from MnSOD^K68Q^ expression, behaves as a tumor promoter [92].

In a more recent study by He et al. analyzed a set of data that indicated how the MnSOD-dependent induction of a cancer stem cell-like phenotype was not actually related to superoxide dismutation but the peroxidase activity associated with MnSOD acetylation [93]. Hjelmeland and Patel imply that it is not the idea that MnSOD expression was higher in cancer that is central, but actually that acetylation of MnSOD was also elevated unexpectedly with higher protein expression. Therefore, He et al. suggest that acetylation of MnSOD is the essential switch converting MnSOD from a dismutase antioxidant to a peroxidase prooxidant and the pro-HIF2α mediator which is linked to higher cancer stem cell maintenance [94].

The regulation of MnSOD activity by phosphorylation was observed initially in *Listeria monocytogenes* and *Campybacter jejuni* [95,96]. MnSOD was shown to be a target of phosphorylation in the mitochondria of the porcine heart [97], potatoes [98] and rats [99]. In human MnSOD, the residue most likely to undergo phosphorylation is S106, lying within the Cdk1 phosphorylation consensus sequence (Ser/Thr/Pro). This was substantiated via the replacement of serine by alanine. MnSOD phosphorylation by the cdk1/cyclin B1 complex doubled MnSOD activity and stabilized the protein’s tetrameric structure [100] conveying a survival advantage to the cell under very stressful conditions. Besides, the augmented hydrogen peroxide production generated by the activated MnSOD promotes transitioning in the cell cycle from G2 to the M phase [101].

Luo et al. suggest that 17β-estradiol-mediated cardioprotection is correlated to the interaction between MnSOD found in the heart and mitochondrial p38β [102]. In this case, the phosphorylation of amino acids T79 and S106 in MnSOD by the kinase is crucial for the suppression of ROS. Overexpression of wild-type MnSOD in cardiomyocytes reduced ROS generation during hypoxia/reoxygenation, while the point mutation of T79 and S106 to alanine in MnSOD abolished its antioxidative function. Luo et al. concluded that the protective effects of 17β-estradiol and the estrogen receptor against cardiac ischemia/reperfusion injury are due to MnSOD regulation via post-translational modifications by p38β [102].

Peroxynitrite (ONOO^-^) is a highly reactive nitrogen species produced from nitric oxide **^.^**NO, and it has been linked to a number of pathological states [103,104] mainly due to the nitration of tyrosine amino acids. The residue which is most susceptible to nitration by peroxynitrite in MnSOD is Y34. Cells are protected by MnSOD which reacts directly with peroxynitrite (Figure 6). The latter process of detoxification, however, inactivates MnSOD [105–108].

Another post-translation modification of MnSOD involves the addition of glutathione and is referred to as glutathionylation. Normal renal proximal tubular cells (NRK) in rats were treated with *S*-nitrosoglutathione which inactivated MnSOD reversibly via the alteration of residue C196 [109].

## Conclusions and perspectives

Although MnSOD is principally involved in providing cells with protection against oxidative stress, this review on the characterization of MnSOD synthetic mutants and natural variants allowed us to explore many other critical roles that ubiquitous MnSODs may play.

The several studies involving reverse engineering of the active site ligands and second-sphere residues in different MnSODs reinforce the essential role played by the hydrogen-bonded network in enzyme catalysis. As different MnSODs have highly comparable active sites, it is difficult to identify the structural features responsible for the changes in MnSOD catalysis between different species, especially with respect to the different product inhibition levels observed. The production of hydrogen peroxide may be regarded as important as the elimination of superoxide anions. High amounts of hydrogen peroxide are toxic; however, the generation of small quantities of hydrogen peroxide as a by-product of MnSOD provides a system in which it can be used in cell signaling pathways.

Post-translational modifications of MnSODs such as acetylation and phosphorylation, provide us with an idea that such modifications in MnSOD must be essential to the cell, and that MnSODs have other functions in addition to simply removing superoxide. This review also highlights studies that have demonstrated how the aberrant expression of MnSOD, such as the predominantly studied Val16Ala polymorphism is implicated in many cancer types [111].

The use of MnSOD mutants in the form of combination therapy may be beneficial in cancer treatment as observed in the case of rMnSOD or in the *in vitro* studies for human H30N MnSOD. Here, modified MnSODs may be designed to actively target tumor cells or may be incorporated into delivery systems such as liposomes to prolong the half-life of MnSOD and simplify cellular uptake. Understanding the structure and catalysis of both wild-type and mutant MnSODs may ultimately serve to synthetically design more stable SOD mimetics, which may be of therapeutic value [7].

## Abbreviations

ETC: electron transport chain
HFEPR: high-field electron paramagnetic resonance
ROS: reactive oxidative species
